# Lower Podocyte Number per Glomerulus Associates With Progressive CKD

**DOI:** 10.1016/j.ekir.2025.06.004

**Published:** 2025-06-11

**Authors:** Aleksandar Denic, Afsana A. Shaik, Syed Khooshal Fareeduddin, Aperna Fnu, Mahesh Kumar, Aidan F. Mullan, Vidit Sharma, Mariam P. Alexander, Akhilesh Pandey, Roger C. Wiggins, Jeffrey B. Hodgin, Stuart J. Shankland, Andrew D. Rule

**Affiliations:** 1 Division of Nephrology and Hypertension, Mayo Clinic, Rochester, Minnesota, USA; 2 Division of Biomedical Statistics and Informatics, Mayo Clinic, Rochester, Minnesota, USA; 3 Department of Urology, Mayo Clinic, Rochester, Minnesota, USA; 4 Department of Laboratory Medicine and Pathology, Mayo Clinic, Rochester, Minnesota, USA; 5 Manipal Academy of Higher Education, Manipal, Karnataka, India; 6 Division of Nephrology, University of Michigan, Ann Arbor, Michigan, USA; 7 Department of Pathology and Internal Medicine, Division of Nephrology, University of Michigan, Ann Arbor, Michigan, USA; 8 Division of Nephrology, University of Washington, Seattle, Washington, USA; 9 Division of Epidemiology, Mayo Clinic, Rochester, Minnesota, USA

**Keywords:** chronic kidney disease, epidemiology, glomerulus, parietal epithelial cell, podocyte, stereology

## Abstract

**Introduction:**

Podocyte depletion may play an important role in chronic kidney disease (CKD); however, longitudinal clinical studies are lacking in populations without obesity-related glomerulopathy.

**Methods:**

Patients studied underwent radical nephrectomy for tumors between 2000 and 2021 and had < 10% interstitial fibrosis and tubular atrophy (IFTA) on histology. Cases who developed progressive CKD were identified by the onset of dialysis, kidney transplantation, sustained estimated glomerular filtration rate (eGFR) < 10 ml/min per 1.73 m^2^, or sustained 30% eGFR decline after the postnephrectomy baseline eGFR. Each case of progressive CKD was age-sex-matched to a control without progressive CKD at the same follow-up time. Podometric measures (number/glomerulus, density, and cell volume) and parietal epithelial cell (PEC) measures (number/glomerulus and density) were determined with immunohistochemistry and stereology. The risk of progressive CKD with podometric measures was assessed, adjusting for glomerular tuft volume, glomerulosclerosis, IFTA, and arteriosclerosis (all determined by morphometry) or adjusting for age, sex, diabetes mellitus, body mass index (BMI), hypertension, eGFR, and proteinuria.

**Results:**

There were 35 CKD cases and 35 controls studied. Cases versus controls did not differ significantly by BMI (mean: 29.1 vs. 28.2 kg/m^2^, *P* = 0.88), glomerular tuft volume, or PEC number, but had lower podocyte number, lower podocyte density, and lower PEC density. Lower podocyte number correlated with several nephrosclerosis measures. In adjusted analyses, podocyte number and podocyte density associated with progressive CKD and these associations did not substantively differ by cortex depth.

**Conclusion:**

Low podocyte number per glomerulus is associated with the development of progressive CKD independent of CKD risk factors, kidney function, glomerular volume, and nephrosclerosis severity.

## Graphical abstract

**Figure:**
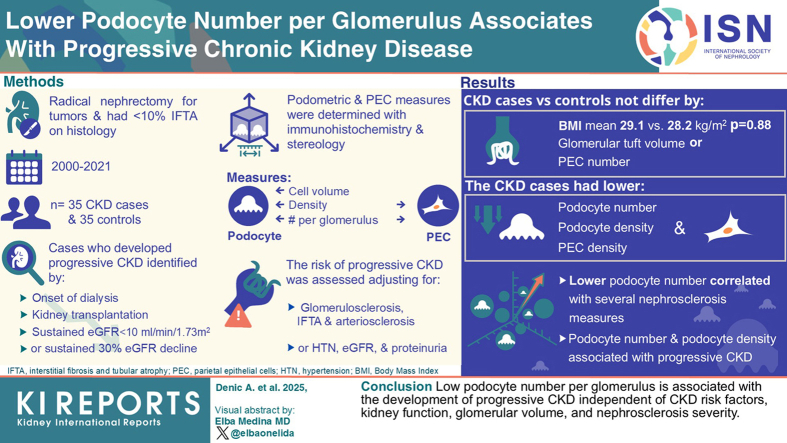

Podocytes are postmitotic epithelial cells that are a crucial component of the glomerular filtration barrier.^1^ In addition to limiting the passage of albumin and other proteins from the blood to the urinary space, podocytes make vascular endothelial growth factor critical for the health of neighboring glomerular endothelial cells. Podocytes are injured in primary podocytopathies, diabetes mellitus, hypertension, and other diseases. The podocyte depletion hypothesis,^2^ explains that following injury, loss of podocytes and compensatory hypertrophy contribute to glomerulosclerosis and persistent proteinuria, ultimately leading to kidney failure regardless of the initial cause of glomerular injury. Podometric measures, particularly reduced podocyte number, have been associated with reduced nephron number.^3^^,^^4^ Wiggins *et al.*^2^ suggested the importance of monitoring podocyte injury and depletion because many glomerular diseases share a common podocyte-dependent mechanism. In animal models and human biopsies, studies showed that podocytes can undergo significant growth (hypertrophy), but only to a certain degree to compensate for podocyte loss.^5^^,^^6^ Current understanding of research is that both compensatory podocyte hypertrophy and podocyte depletion, whether absolute or relative, are early events in the progression of glomerulosclerosis.^2^^,^^7^^,^^8^ The potential role of PECs has recently emerged. Under certain circumstances, a subpopulation of PECs may be able to partially replace adult podocytes that have been depleted in disease.^9^ In contrast, when PECs acquire an activated phenotype characterized as profibrotic and migratory, their increased production of extracellular matrix proteins leads to glomerulosclerosis.^10^^,^^11^

To our knowledge, only 1 study has investigated the long-term risk of progressive CKD with podometric measures. Haruhara and colleagues found that lower podocyte density (but not lower podocyte number/glomerulus) was predictive of progressive CKD among patients with obesity-related glomerulopathy.^12^ Whether these findings are generalizable to other patient populations is unclear. Further, the importance of PEC number and of podometric measures at different depths in the kidney cortex to the risk of progressive CKD is unknown. Patients who undergo a radical nephrectomy for tumor with availability of large tissue wedge sections provide a unique opportunity to study podometric measures in a population at high-risk for progressive CKD.^13^ We have previously found that in the superficial cortex, glomeruli are smaller compared with the deeper cortex.^14^ Thus, we studied podometric measures across different depths in the cortex. The goal of this study was to determine if podometric measures associated with the subsequent development of progressive CKD among patients who underwent a radical nephrectomy and had none to mild (< 10%) IFTA on histology.

## Methods

### Study Design

We studied patients in the Aging Kidney Anatomy study^15^ who had undergone a radical nephrectomy for a renal tumor at the Mayo Clinic, Rochester, Minnesota between 2000 and 2021, and who had no metastatic lesions or positive lymph nodes at the time of surgery. These patients were followed-up every 3 to 6 months for the first year and then every 6 to 12 months (via a standardized clinical protocol to evaluate for cancer recurrence or CKD). The last follow-up in this study was on September 1, 2023. We also excluded patients if they lacked a baseline serum creatinine test between the nephrectomy and 4 months after the nephrectomy, lacked a follow-up serum creatinine at least 4 months after the nephrectomy, had cancer recurrence, death, kidney failure during the first 4 months after their nephrectomy, had a specific glomerular diseases such as amyloidosis or Ig A nephropathy (mild-moderate diabetic nephropathy was allowed—up to class II but not class III, IV, or V),^16^ had severe and diffuse tubulointerstitial inflammation, or had cortical IFTA ≥ 10% by morphometry. In this study, cases with progressive CKD were identified by a ≥ 30% decline in eGFR from a postnephrectomy baseline eGFR that was sustained for at least 3 months, an eGFR < 10 ml/min per 1.73 m^2^ and at least 5 ml/min per 1.73 m^2^ below postnephrectomy baseline eGFR that was sustained for at least 3 months, dialysis, or kidney transplantation. Progressive CKD was required to be documented before any cancer recurrence beyond the first 4 months postnephrectomy. Of the remaining patients, age, sex, and follow-up time matched controls without progressive CKD were identified for each case. Cases and controls were also required to have formalin-fixed paraffin-embedded tissue blocks available for obtaining sections for immunohistochemistry. This study was approved by the Mayo Clinic Institutional Review Board with a waiver of consent because data were limited to the medical record and specimens obtained for clinical care.

### Clinical Characteristics

From the medical records we obtained prenephrectomy clinical characteristics (age, sex, height, weight, BMI, serum creatinine [corrected to standardized values if assayed prestandardization], and 24-hour urine protein. Diagnoses of hypertension and diabetes mellitus were based on review of notes in the medical records. The number of antihypertensive medications and use of angiotensin-converting enzyme inhibitors or angiotensin receptor blockers was also determined. All patients with diabetes had their histology reviewed and scored for diabetic nephropathy.^16^ eGFR was calculated using the 2021 CKD Epidemiology Collaboration serum creatinine–based equation.^17^ If there were multiple serum creatinine values in the first 4 months postnephrectomy, we chose the one closest to but before 4 months after the nephrectomy as the baseline eGFR. We estimated the 24-hour urine protein excretion from a spot urine protein-osmolality ratio.^18^ If a postnephrectomy level was not obtained within the first 4 months, a prenephrectomy urine protein was used.

### Detection of Podocytes and PECs

**Figure 1:**
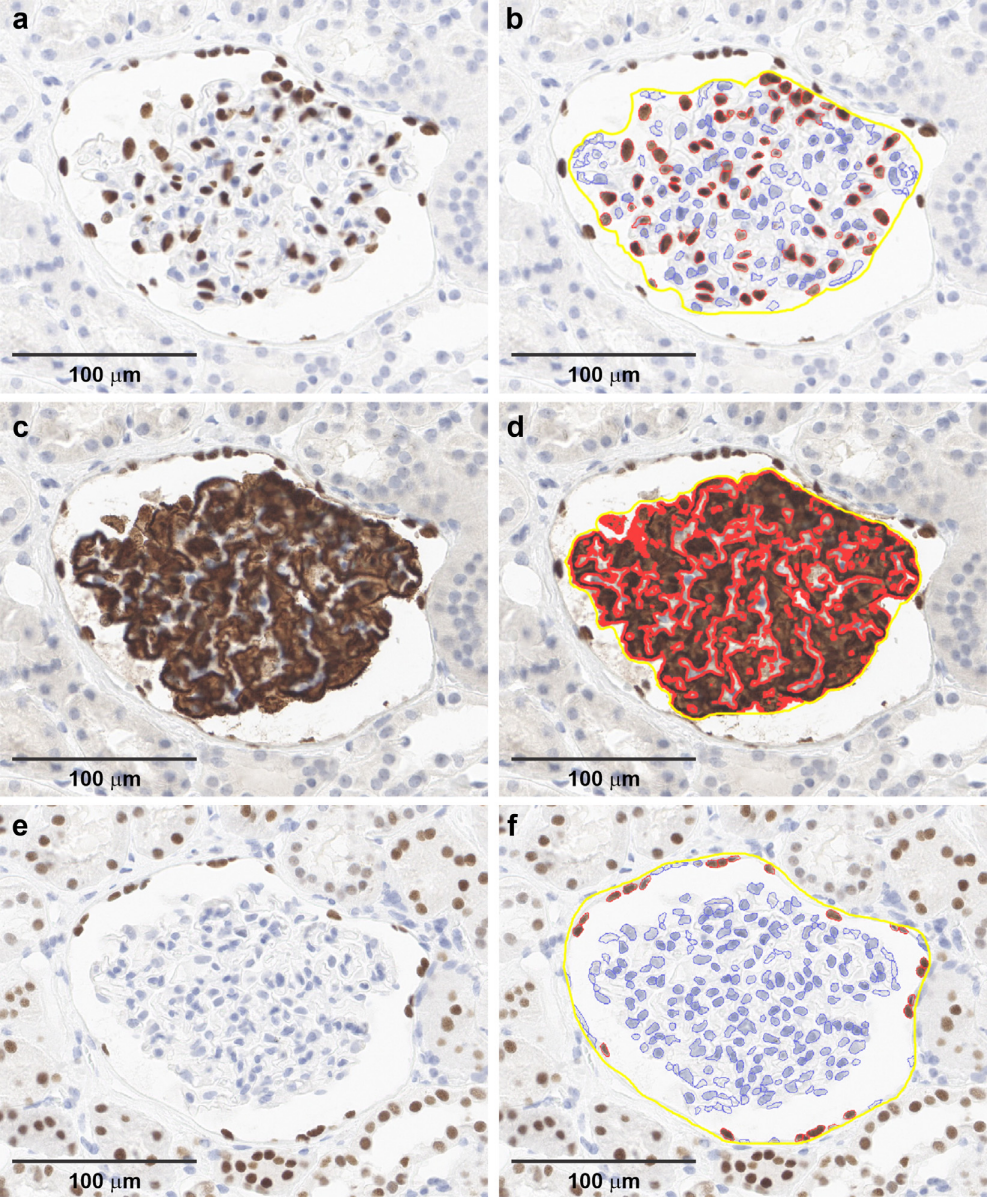
Example of immunohistochemistry staining in the same glomerular section for (a and b) visceral podocyte nuclei (WT1) and then (c and d) overstained for podocyte cytoplasm (GLEPP1). Adjacent section of the same glomerulus stained for (e and f) parietal epithelial cells (PAX8). Left panel is the raw image and right panel is the segmentation within the annotated yellow boundary for nuclei (red outline in b and f) and cytoplasm (red outline in d) using QuPath.

Each patient had 2 consecutive wedge sections of kidney tissue away from the tumor evaluated with podometrics after undergoing immunohistochemistry staining (Supplementary Methods). We estimated each section’s thickness under oil immersion microscopy at 1000× by measuring the distance between top and bottom edges of the section using the calibrated focusing dial on the microscope.^19^ Podocyte nuclei were identified by WT1^20^ staining located within the glomerular tuft (Figure 1a and b). WT1-positive nuclei located on the Bowman’s capsule (PECs) were not counted as podocytes.^21^ After obtaining a 400× digital image of the WT1-stained section, podocyte cytoplasm was identified by restaining with GLEPP1^22^ staining on the same glomerular tuft (Figure 1 c and d) and a second 400× digital image of the same section was obtained. Finally, on the second section, PEC nuclei were identified by PAX8^23^ staining located on the Bowman’s capsule (Figure 1e and f) and a third 400× digital image was obtained. These 3 digital images were analyzed in a random order masked to case or control status, clinical characteristics, and outcomes. Glomerular profiles within 1 mm of the kidney capsule were considered superficial depth and those within 1 mm of the corticomedullary junction were considered to be deep. A parallel curve was drawn at low magnification approximately halfway between the kidney capsule and corticomedullary junction, and glomeruli within 1 mm of this curve were considered at middle depth (Supplementary Figure S1).

### PEC Nuclear Shape Coefficient

A nuclear shape coefficient accounts for the extent a nucleus deviates from a perfect sphere (k = 0.79) and has been derived for podocytes (k = 0.72)^19^ but is unknown in PECs. To determine the PEC shape coefficient, we obtained 4 sections of different thicknesses (3, 5, 7, and 9 μm were targeted but the true diameter of each section was determined with oil immersion microscopy at 1000×) for 1 case and 1 control. Each was stained for PAX8 (for PEC nuclei) and scanned into digital images. For each image, PAX8-stained nuclei lining the inside of the Bowman’s capsule profile were counted and their mean caliper diameter calculated using QuPath. Using the formula by Venkatareddy *et al.*^19^ that calculates true nuclear caliper diameter (D) from observed nuclear caliper diameter (d), tissue thickness (T), and the nuclear shape coefficient (k) (Equation 1), we performed a nonlinear fit across the different tissue thicknesses to determine this shape coefficient for PEC nuclei, which was 0.62 for the case and 0.60 for the control. Thus, we used k = 0.61 for the PEC nuclei shape coefficient. Profiles of podocyte and PEC nuclei on toluidine blue stained sections on electron microscopy are shown in Supplementary Figure S2.

$$\text{Equation 1:}\;\text{True}\;\text{nuclear}\;\text{caliper}\;\text{diameter}\;\left(D\right)=\frac{d-T+\sqrt{{\left(d-T\right)}^{2}-4\text{kdT}}}{2k}$$

### Podometrics

We characterized podocytes and PEC in nonsclerosed glomeruli at each of the 3 depths. Glomerular tuft profiles within 1 mm of the superficial, middle, and deep depths were traced (Supplementary Figure S1). The median (range) of traced glomeruli at superficial, middle, and deep depths were 21 (11–33), 27 (16–38), and 25 (16–38), respectively. In QuPath (version 0.5.1), all WT1-stained nuclei within the glomerular tuft were counted and their observed caliper diameter (d) was converted to a true nuclear caliper diameter (D) (Equation 1). The true podocyte nuclear number per glomerular tuft section was determined (Equation 2).^19^ Finally, podocyte density (per mm^3^) was calculated (Equation 3).^19^

$$\text{Equation 2:}\;\text{True}\;\text{podocyte}\;\text{nuclear}\;\text{number}=\frac{1}{\frac{D}{T}+1}\times \text{Number}\;\text{of}\;\text{observed}\;\text{podocytes}\;\text{per}\;\text{glomerular}\;\text{tuft}$$

$$\text{Equation 3:}\;\text{Podocyte}\;\text{Density}=T\times \text{Glomerular}\;\text{tuft}\;\text{area}\;\left({\mu m}^{2}\right)\times \text{True}\;\text{podocyte}\;\text{nuclear}\;\text{number}$$

On WT1-Glepp1 double-stained scanned images, the same glomerular tufts at all 3 depths were analyzed. Using QuPath, the WT1-Glepp1 positive areas identified the total podocyte cell area for each glomerular tuft profile. We calculated the %podocyte cell area of each tuft profile by dividing the podocyte cell area by the glomerular tuft profile area for each glomerulus, which also represents %podocyte cell volume of the tuft volume.^24^ On PAX8-stained scanned images, we traced the capsules of the adjacent profiles of the glomeruli as used for the WT1-Glepp1 double-stained slides. Using QuPath, all PAX8-stained nuclei were counted and their mean caliper diameter calculated. The true PEC nuclear number per glomerular profile was then estimated (Equation 1),^19^ but using the newly derived shape coefficient of 0.61 for PEC nuclei. The PEC density (per area of capsule in μm^2^) was then calculated.^19^

$$\text{Equation 4:}\;\text{PEC}\;\text{Density}=\frac{\text{True}\;\text{PEC}\;\text{nuclear}\;\text{number}}{\text{Mean}\;\text{capsular}\;\text{circumference}\times T}$$

### Converting Podocyte and PEC Density to Number per Glomerulus

Using the Weibel and Gomez formula,^25^ the glomerular volume at the tuft level and at the Bowman’s capsule level was calculated from the mean area of the glomerular tuft and capsule profiles for each cortical depth. The capsule volume was converted to a capsule surface area based on the formula for a sphere. At each depth, the podocyte number per glomerulus was calculated by multiplying podocyte density by glomerular tuft volume. At each depth, the podocyte cell volume was calculated as follows: (glomerular tuft volume × %podocyte cell volume)/podocyte number per glomerulus. At each depth, PECs per glomerulus were calculated by multiplying PEC area density by glomerular capsule surface area. The mean of the 3 depths was used to calculate the overall podocyte and PEC number per glomerulus.

### Morphometric Measures of Nephrosclerosis and Nephron Number

Nephrosclerosis measures by morphometry included the %globally sclerosed glomeruli, %IFTA, IFTA foci density, and %luminal stenosis from intimal thickening in arteries as previously described.^26^^,^^27^ These measures were calculated from the entire cortex of the wedge sections. Nephron number was calculated from stereological estimates of 3-dimensional nonsclerosed glomerular density on histology and cortical volume on contrast enhanced image (computed tomography or magnetic resonance imaging) as previously reported.^15^

### Statistical Analyses

Clinical characteristics and kidney histology measures were compared between cases and controls using paired Wilcoxon signed-rank tests and exact McNemar’s tests as appropriate. Nonparametric Spearman’s correlations were used to compare podometric measures with each other and with clinical characteristics, glomerular tuft volume, and measures of nephrosclerosis (%globally sclerosed glomeruli, %IFTA, IFTA foci density, and %luminal stenosis). A conditional logistic regression model accounting for the matched case-control design was used to assess the risk of progressive CKD with podometric measures. To allow meaningful comparisons of effect sizes (odds ratios), all podometric measures were standardized to per SD increments. Models were unadjusted, adjusted for histological measures, or adjusted for clinical characteristics. For the primary analysis, least absolute shrinkage and selection operator regression was used to select the histological measures or the clinical characteristics to include as covariates in the adjusted analyses. However, as a sensitivity analyses, all histological measures (glomerular tuft volume, %globally sclerosed glomeruli, %IFTA, IFTA foci density, and %artery luminal stenosis) or all clinical characteristics (diabetes mellitus, hypertension, BMI, baseline eGFR, and proteinuria) were adjusted for in the models. Sensitivity analyses were performed using unconditional logistic regression, excluding patients with diabetes mellitus or diabetic nephropathy. Statistical analyses were performed using BlueSky Statistics software version 10.3.4 (BlueSky Statistics LLC, Chicago, IL) and R (RStudio) version 4.3.2.

## Results

**Figure 2:**
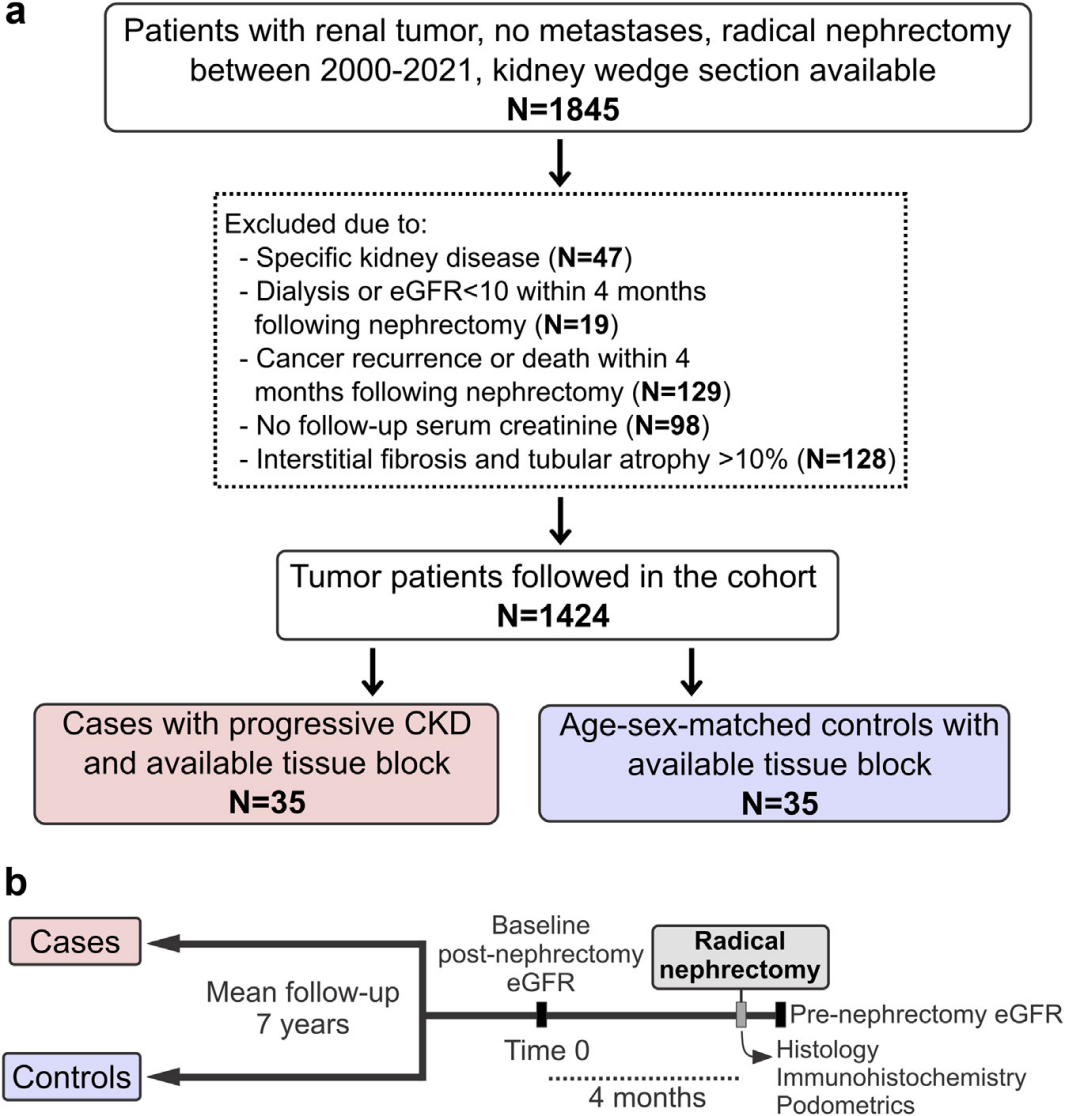
Nested case-control study design. (a) Sample size during selection of cases and controls. (b) Timing of cases and controls relative to baseline measures.

From a cohort of 1845 patients who underwent a radical nephrectomy for tumor, we identified 35 cases who developed progressive CKD and matched them on age (mean: 67.4 years), sex (74% male), and follow-up time (mean: 6.8 years) to 35 controls who did not develop progressive CKD (Figure 2). There were a mean ± SD of 16 ± 17 eGFR tests among cases and 11 ± 13 eGFR tests among controls during follow-up until censoring. Of the 35 cases, 1 underwent kidney transplantation, 3 started dialysis, 8 had sustained eGFR < 10ml/min per 1.73 m^2^, and 23 had sustained >30% eGFR decline. Baseline clinical and histology characteristics of cases and controls are shown in Table 1. Cases compared with controls had more diabetes mellitus and higher baseline eGFR but otherwise did not significantly differ in baseline clinical characteristics or in follow-up time. Of the 11 patients with diabetes mellitus, 4 had class IIa diabetic nephropathy (mild mesangial expansion) and 1 had class IIb (moderate mesangial expansion). During follow-up, cases had a mean of 33.7% decrease in eGFR, and controls had a mean of 9.1% increase in eGFR. Notably, glomerular tuft volume and glomerular capsule surface area did not differ between cases and controls, overall or at any depth. Glomeruli were smallest in the superficial cortex. Cases had more glomerulosclerosis and IFTA on histology than controls.

**Table 1:** Clinical and histology characteristics in cases with progressive CKD compared with matched controls

Characteristics	Progressive CKD (*n* = 35)	Controls (*n* = 35)	*P* value
Clinical characteristics			
Age, yrs	67.4 (11.1)	66.2 (9.8)	0.72
Male	22 (73.7%)	22 (73.7%)	1.00
Weight, kg	83.3 (22.6)	83.0 (17.3)	0.58
BMI, kg/m^2^	29.1 (6.2)	28.2 (5.5)	0.88
Diabetes mellitus	9 (25.7%)	2 (5.7%)	0.04
Hypertension	28 (80.0%)	23 (65.7%)	0.21
Number of antihypertensive medications	1.6 (1.2)	2.0 (0.9)	0.23
Use of ACE inhibitors/Angiotensin receptor blockers	16 (45.7%)	17 (48.6%)	0.74
24-h urine protein, mg	221 (75–456)	96 (60–181)	0.62
Prenephrectomy eGFR, ml/min per 1.73 m^2^	69.4 (12.6)	73.6 (19.7)	0.26
Baseline postnephrectomy eGFR, ml/min per 1.73 m^2^	56.0 (14.2)	50.5 (8.7)	0.04
Follow-up time^a^, yrs	6.8 (6.3)	7.6 (7.4)	0.77
eGFR at follow-up^a^, ml/min per 1.73 m^2^	37.4 (26.4)	54.8 (11.3)	0.0008
Change in eGFR at follow-up^a^, %	–33.7% (45.0)	+9.1% (18.5)	< 0.0001
Histological measures			
Nephron number per kidney^b^, in millions	0.93 (0.39)	1.04 (0.40)	0.52
Glomerular tuft volume overall, mm^3^	0.0032 (0.0011)	0.0031 (0.0010)	0.45
Glomerular tuft volume at superficial depth, mm^3^	0.0028 (0.0009)	0.0027 (0.0011)	0.42
Glomerular tuft volume at middle depth, mm^3^	0.0036 (0.0014)	0.0034 (0.0013)	0.79
Glomerular tuft volume at deep depth, mm^3^	0.0033 (0.0013)	0.0031 (0.0009)	0.36
Glomerular capsule surface area overall, μm^2^	0.14 (0.03)	0.14 (0.03)	0.17
Glomerular capsule surface area at superficial depth, mm^2^	0.13 (0.03)	0.13 (0.03)	0.16
Glomerular capsule surface area at middle depth, mm^2^	0.15 (0.03)	0.15 (0.03)	0.44
Glomerular capsule surface area at deep depth, mm^2^	0.14 (0.04)	0.13 (0.02)	0.07
Globally sclerosed glomeruli, %	12.9 (12.3)	6.7 (4.1)	0.004
Interstitial fibrosis/tubular atrophy, %	3.2 (2.5)	1.4 (1.3)	< 0.0001
Interstitial fibrosis/tubular atrophy foci density, /cm^2^	37.0 (24.3)	22.9 (15.7)	0.02
Artery luminal stenosis, %	56.9 (14.6)	50.1 (15.7)	0.12

ACE, angiotensin-converting enzyme; BMI, body mass index; CKD, chronic kidney disease; eGFR, estimated glomerular filtration rate; IQR, interquartile range.

Data shown as Mean (SD), N (%), or Median (IQR).

a Follow-up was the time the case developed progressive CKD, and the control did not and had a follow-up eGFR. If a case was on dialysis or underwent a kidney transplant, their follow-up eGFR was set to 0 ml/min per 1.73 m^2^.

b Nephron number available in 23 case-control pairs.

The correlations of baseline clinical and histological characteristics with overall podometrics using the combined 35 cases and 35 controls is shown in Table 2. Larger glomerular tuft volume correlated with higher BMI and hypertension. Lower podocyte number correlated with male, higher %globally sclerosed glomeruli, higher %IFTA, and higher IFTA foci density. Lower podocyte density correlated with hypertension. Larger podocyte cell volume correlated with hypertension and proteinuria and lower nephron number. Higher PEC number per glomerulus correlated with younger age, taller height, and higher prenephrectomy eGFR. Larger glomerular tuft volume was correlated with lower podocyte density, larger podocyte cell volume, higher PEC number, and lower PEC density.

**Table 2:** Spearman’s correlations of baseline clinical and histological characteristics with overall podometrics (averaged across depths) among the combined 35 cases and 35 controls

Baseline characteristics	Glomerular tuft volume		Podocyte number per glomerulus		Podocyte density		Podocyte cell volume		PEC number per glomerulus		PEC density	
Clinical characteristics	R_s_	*P* value	R_s_	*P* value	R_s_	*P* value	R_s_	*P* value	R_s_	*P* value	R_s_	*P* value
Age	−0.16	0.19	−0.21	0.09	−0.04	0.74	0.13	0.29	−0.30	0.01	−0.18	0.15
Male	0.06	0.64	−0.33	0.005	−0.22	0.07	0.16	0.20	0.17	0.15	0.10	0.43
Height	0.17	0.16	−0.10	0.40	−0.16	0.19	0.17	0.17	0.33	0.005	0.21	0.08
Body mass index	0.30	0.01	−0.02	0.87	−0.20	0.09	0.18	0.14	0.06	0.65	−0.11	0.38
Diabetes mellitus	0.18	0.13	−0.11	0.37	−0.14	0.23	0.17	0.16	−0.08	0.50	−0.19	0.11
Hypertension	0.30	0.01	−0.16	0.18	−0.33	0.005	0.38	0.001	0.02	0.84	−0.19	0.12
Use of ACE inhibitors or angiotensin receptor blockers^a^	0.14	0.34	0.15	0.28	0.09	0.55	−0.13	0.37	−0.02	0.88	0.01	0.94
Prenephrectomy eGFR	0.11	0.36	0.11	0.35	0.06	0.62	0.01	0.97	0.29	0.01	0.14	0.24
Proteinuria	0.24	0.09	−0.11	0.47	−0.20	0.18	0.34	0.02	0.27	0.06	0.15	0.30
Histological measures												
Glomerular tuft volume	-	-	0.16	0.20	−0.68	<0.0001	0.68	<0.0001	0.28	0.02	−0.30	0.01
Nephron number^b^	−0.23	0.09	−0.06	0.67	0.15	0.28	−0.32	0.02	0.12	0.38	0.23	0.09
% globally sclerosed glomeruli	−0.04	0.71	−0.27	0.03	−0.16	0.18	0.14	0.25	−0.21	0.07	−0.16	0.19
% interstitial fibrosis/tubular atrophy	0.02	0.88	−0.24	0.04	−0.14	0.26	0.09	0.44	−0.12	0.33	−0.18	0.13
Interstitial fibrosis or tubular atrophy foci density	−0.19	0.11	−0.24	0.04	0.04	0.70	−0.07	0.58	−0.14	0.26	−0.05	0.68
%Artery luminal stenosis	0.10	0.39	−0.10	0.39	−0.11	0.37	0.20	0.09	−0.03	0.79	−0.06	0.62

ACE, angiotensin converting enzyme; eGFR, estimated glomerular filtration rate; PEC, parietal epithelial cell; R_s_, Spearman’s correlation coefficient.

a Analyzes limited to 51 patients with hypertension.

b Nephron number was available in 57 patients.

Both the podocyte number per glomerulus and podocyte density were significantly lower in cases with progressive CKD than in controls, both overall and at each cortex depth (Table 3). The podocyte cell volume and PEC number per glomerulus was not significantly different between cases and controls, overall or at any of the 3 cortex depths. The PEC density was lower in cases than in controls, overall and in the middle and deep cortex. In analysis adjusting for %IFTA (selected based on least absolute shrinkage and selection operator regression), both lower podocyte number per glomerulus and lower podocyte density associated with progressive CKD, and these associations remained significant at the deep region (Table 4). In analysis adjusting for diabetes mellitus and baseline eGFR (selected based on least absolute shrinkage and selection operator regression), only lower podocyte number per glomerulus remained significantly associated with progressive CKD, overall and in the middle and deep cortex. In sensitivity analyses adjusting for glomerular volume and all nephrosclerosis measures or adjusting for all clinical characteristics, the findings were not substantively different (Supplementary Table S1). In another set of sensitivity analyses excluding patients with diabetic mellitus (*n* = 11) or excluding patients with diabetic nephropathy (by histology) (*n* = 5), lower podocyte number per glomerulus, overall and in deep regions remained associated with progressive CKD (Supplementary Table S2). In analyses adjusting podocyte number per glomerulus, podocyte cell volume, and PEC density for each other, only lower podocyte number per glomerulus associated with progressive CKD (Supplementary Table S3). When podocyte density replaced podocyte number per glomerulus in the model, none of the podometric measures had a statistically significant association with progressive CKD (Supplementary Table S3).

**Table 3:** Podometrics overall and by cortex depth in cases who develop progressive CKD versus controls

Podometric measurements	Cases who developed progressive CKD (*n* = 35)	Controls (*n* = 35)	*P* value
Overall cortex			
Podocyte number/glomerulus	424 (381–507)	500 (456–551)	0.009
Podocyte density, /10^6^ mm^3^	139 (105–183)	165 (143–211)	0.01
Podocyte cell volume, mm^3^	3342 (2883–3930)	3114 (2809–3744)	0.30
PEC number/glomerulus	242 (193–301)	267 (220–316)	0.29
PEC density, /mm^2^	0.0018 (0.0013–0.0021)	0.0021 (0.0017–0.0023)	0.03
Glomerular tuft volume, mm^3^	0.0032 (0.0011)	0.0031 (0.0010)	0.55
Superficial cortex			
Podocyte number /glomerulus	447 (361–518)	502 (437–587)	0.04
Podocyte density, /10^6^ mm^3^	152 (112–214)	195 (149–267)	0.02
Podocyte cell volume, mm^3^	3075 (2,391–3,754)	2717 (2,378–3,426)	0.32
PEC number /glomerulus	275 (189–339)	253 (207–316)	0.76
PEC density, /mm^2^	0.0020 (0.0016–0.0023)	0.0022 (0.0018–0.0027)	0.17
Glomerular tuft volume, mm^3^	0.0028 (0.0009)	0.0027 (0.0011)	0.71
Middle cortex			
Podocyte number /glomerulus	420 (378–485)	485 (425–564)	0.02
Podocyte density, /10^6^ mm^3^	122 (94–168)	157 (122–194)	0.02
Podocyte cell volume, mm^3^	3703 (3,174–4,351)	3416 (3,028–4,150)	0.31
PEC number /glomerulus	257 (176–306)	275 (227–331)	0.20
PEC density, /mm^2^	0.0017 (0.0013–0.0021)	0.0020 (0.0015–0.0024)	0.03
Glomerular tuft volume, mm^3^	0.0036 (0.0014)	0.0034 (0.0013)	0.63
Deep cortex			
Podocyte number /glomerulus	394 (312–489)	499 (421–550)	0.004
Podocyte density, /10^6^ mm^3^	137 (105–168)	155 (136–206)	0.02
Podocyte cell volume, mm^3^	3428 (3,118–4,218)	3487 (2,877–3,939)	0.27
PEC number /glomerulus	244 (169–296)	270 (199–307)	0.28
PEC density, /mm^2^	0.0016 (0.0013–0.0021)	0.0021 (0.0016–0.0023)	0.03
Glomerular tuft volume, mm^3^	0.0033 (0.0013)	0.0031 (0.0009)	0.41

CKD, chronic kidney disease; IQR, interquartile range; PEC, parietal epithelial cell.

Data shown as Mean (SD) or Median (IQR).

**Table 4:** Risk of progressive CKD with podocyte measures

Podometrics (all per SD)	Unadjusted	Adjusted for %IFTA^a^	Adjusted for eGFR and diabetes mellitus^b^
	OR (95% CI)	OR (95% CI)	OR (95% CI)
Overall cortex			
Podocyte number per glomerulus	0.39 (0.19–0.79)	0.42 (0.18–0.98)	0.31 (0.13–0.77)
Podocyte density	0.54 (0.30–0.96)	0.47 (0.22–0.97)	0.55 (0.27–1.10)
Podocyte cell volume	1.48 (0.87–2.52)	1.64 (0.79–3.41)	1.41 (0.78–2.55)
PEC number per glomerulus	0.87 (0.54–1.40)	0.95 (0.49–1.84)	0.76 (0.41–1.43)
PEC density	0.69 (0.42–1.15)	0.66 (0.35–1.24)	0.69 (0.37–1.31)
Superficial cortex			
Podocyte number per glomerulus	0.54 (0.29–0.98)	0.62 (0.30–1.28)	0.55 (0.26–1.19)
Podocyte density	0.55 (0.31–0.98)	0.55 (0.29–1.06)	0.43 (0.18–1.02)
Podocyte cell volume	1.46 (0.86–2.45)	1.51 (0.76–2.99)	1.45 (0.80–2.63)
PEC number per glomerulus	1.13 (0.71–1.81)	1.44 (0.71–2.92)	1.24 (0.69–2.22)
PEC density	0.82 (0.49–1.35)	0.91 (0.48–1.69)	0.85 (0.44–1.62)
Middle cortex			
Podocyte number per glomerulus	0.43 (0.21–0.89)	0.43 (0.17–1.04)	0.33 (0.14–0.88)
Podocyte density	0.51 (0.28–0.96)	0.38 (0.15–0.93)	0.56 (0.28–1.13)
Podocyte cell volume	1.44 (0.82–2.55)	1.78 (0.74–4.26)	1.37 (0.75–2.53)
PEC number per glomerulus	0.79 (0.49–1.26)	0.77 (0.42–1.43)	0.67 (0.36–1.28)
PEC density	0.68 (0.42–1.11)	0.58 (0.31–1.10)	0.71 (0.39–1.29)
Deep cortex			
Podocyte number per glomerulus	0.34 (0.16–0.72)	0.40 (0.17–0.92)	0.30 (0.12–0.73)
Podocyte density	0.56 (0.32–0.98)	0.49 (0.25–0.94)	0.65 (0.35–1.20)
Podocyte cell volume	1.65 (0.93–2.94)	1.89 (0.81–4.40)	1.53 (0.79–2.99)
PEC number per glomerulus	0.81 (0.47–1.40)	0.87 (0.41–1.82)	0.56 (0.26–1.20)
PEC density	0.66 (0.38–1.12)	0.61 (0.32–1.17)	0.61 (0.32–1.18)

CI, confidence interval; CKD, chronic kidney disease; eGFR, estimated glomerular filtration rate; IFTA, interstitial fibrosis and tubular atrophy; LASSO, least absolute shrinkage and selection operator, PEC, parietal epithelial cell.

a Of histological measures, only %IFTA was selected as a predictor of progressive CKD by LASSO regression.

b Of clinical characteristics, only baseline eGFR and diabetes mellitus were selected as predictors of progressive CKD by LASSO regression.

We assessed the correlation of podocyte number per glomerulus with other podometric measures (Table 5). Lower podocyte number per glomerulus had moderate correlations with lower podocyte density, larger podocyte cell volume, lower PEC number, and lower PEC density. Lower podocyte density, which reflects the ratio of podocyte number per glomerulus with glomerular volume, had stronger correlations with larger podocyte cell volume and with lower PEC density.

**Table 5:** Correlation between different podometric measures (averaged across depths) among the combined 35 cases and 35 controls

Podometrics	Podocyte number per glomerulus		Podocyte density	
	R_s_	*P* value	R_s_	*P* value
Podocyte density	0.52	< 0.0001	---	---
Podocyte cell volume	−0.35	0.003	−0.83	< 0.0001
PEC number per glomerulus	0.49	0.005	0.16	0.20
PEC density	0.41	0.0005	0.59	0.0003

PEC, parietal epithelial cell; R_s_, Spearman’s correlation coefficient.

## Discussion

**Figure 3:**
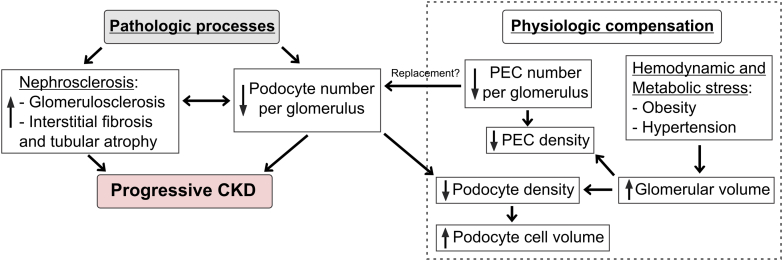
Conceptual diagram of podometric relationships and their association with progressive CKD. Decreased podocyte number per glomerulus associated with progressive CKD, and though it also associated with nephrosclerosis, the association with progressive CKD was also independent of nephrosclerosis. Whereas podocyte number per glomerulus correlated with the parietal epithelial cell (PEC) number per glomerulus, PEC number per glomerulus did not associate with progressive CKD. Although decreased podocyte density was associated with progressive CKD, glomerular volume did not, and this association is via the effects of decreased podocyte number on podocyte density. Thus, there appears to be pathological processes that lead to loss of podocytes and this leads to progressive CKD. Larger glomerular volume does directly decrease podocyte and PEC density with compensatory podocyte cell volume but was not associated with progressive CKD in this setting suggesting a more physiological compensatory process. CKD, chronic kidney disease.

This case-control study compared baseline podometrics between patients who underwent a radical nephrectomy for tumor and then subsequently did (case) or did not (control) develop progressive CKD. Lower podocyte number per glomerulus is associated with progressive CKD, regardless of cortical depth and independent of clinical factors or histological pathology. Lower podocyte density is also associated with progressive CKD but via lower podocyte number per glomerulus rather than glomerular hypertrophy. Lower podocyte number per glomerulus was also the only podometric finding associated with glomerulosclerosis and IFTA on histology. Podocyte loss is thought to be a mechanism for global glomerulosclerosis,^28^ which then leads to IFTA of the attached downstream tubule. There was evidence that glomerular hypertrophy leads to decreased podocyte density and hypertrophy of podocyte cell volume. Podocyte number per glomerulus also correlated with PEC number per glomerulus. However, neither larger glomerular volume nor low PEC number per glomerulus associated with progressive CKD, suggesting that these factors are likely reflective of physiological compensation rather than pathology in this setting (Figure 3). We speculate that other unidentified pathological processes led to the lower podocyte number per glomerulus (possibly via podocyte shedding) associated with progressive CKD. These processes were unidentified in our cohort and patients with specific kidney diseases were excluded from evaluation. The association between lower podocyte number per glomerulus and progressive CKD remained stable despite adjustments for common CKD risk factors, kidney function, glomerular volume, or nephrosclerosis.

The increased risk of progressive CKD with decreased podocyte number per glomerulus is consistent with findings in animal models. Experimentally induced podocyte depletion in transgenic rats demonstrated that progression of kidney disease is directly proportional to the amount of podocyte depletion; loss of > 40% of podocytes led to glomerulosclerosis and a significant decrease in renal function.^29^ A recent study of 46 patients from Japan with obesity-related glomerulopathy found that lower podocyte density (but not podocyte number per glomerulus) associated with progressive CKD, even after adjusting for clinicopathologic risk factors.^12^ The mean glomerular tuft volume of these patients with a mean BMI of 28.8 kg/m^2^ was 0.0036 mm^3^ compared with 0.0032 mm^3^ in this present study of predominately White American patients with kidney tumor with a mean BMI of 29.1 kg/m^2^. Notably, criteria for obesity in a Japanese population is a BMI > 25 kg/m^2^ rather than > 30 kg/m^2^. Reasons for the discrepancy between the 2 studies with respect to podocyte number per glomerulus may be related to the specific pathology of obesity-related glomerulopathy. In this condition, glomerular hypertrophy is thought to overwhelm physiological podocyte compensatory pathways (including podocyte cell hypertrophy).^12^ Considering that podocyte density is inversely associated with glomerular hypertrophy, lower podocyte density is a stronger predictor of progressive CKD in obesity-related glomerulopathy. A decrease in podocyte density can also be due to glomerular tuft hypertrophy from glomerular hyperfiltration in response to nephron loss in intrinsic kidney disease.^30^ It is recognized that elevation in blood pressure leads to glomerular hypertension, which has been suggested to cause podocyte damage.^31^^,^^32^

In this current study, we did not detect a difference in glomerular volume between the 35 cases who developed progressive CKD compared with the 35 matched controls. We were likely underpowered to detect such an association with this nested case-control study because in the full cohort, larger glomerular volume does predict progressive CKD.^33^ However, this lack of difference in glomerular volume between the cases and controls, even if serendipitous, allowed us to isolate the impact of podocyte number per glomerulus on the risk of progressive CKD without the confounding impact of glomerular hypertrophy.

Higher podocyte cell volume is strongly associated with lower podocyte density, supporting the hypothesis that podocyte hypertrophy occurs to maintain the glomerular filtration barrier to compensate for podocyte depletion, though the capacity to do this is limited.^2^^,^^5^^,^^6^ In the current study, larger podocytes associated with both proteinuria and hypertension. Podocytes are terminally differentiated and thus are thought to be unable to self-renew. Podocytes and PECs are derived from the same developmental lineage.^34^ Therefore, factors that result in reduced podocyte cell number per glomerulus could affect the PEC number per glomerulus in parallel, as was observed in this report. Nevertheless, under certain conditions involving podocyte loss, a subset of PECs have been reported to have the capacity to transition into podocyte-like structures.^35^, ^36^, ^37^, ^38^, ^39^ According to this hypothesis, the stem cell–like property of a PEC subpopulation (presumably common precursors to both PECs and podocytes) allows them to repeatedly enter and exit the cell cycle making them potential precursors for podocytes in mature glomeruli.^40^^,^^41^ In the current study, podocyte number per glomerulus correlated with PEC number per glomerulus. This result could be viewed as consistent with the hypothesis that PECs serve as a reservoir for replacing podocytes by suggesting that a reduced PEC number resulted in a reduced podocytes number. Alternatively, one could argue that the fact that the major finding in this present study (lower podocyte number per glomerulus was independently related to glomerulosclerosis and progressive CKD) suggests that this theoretical replenishment capacity of podocytes from the PEC (or any other) population is limited, at least under the conditions examined. The conclusion that podocyte replenishment capacity is limited would also be consistent with the extensive body of data linking podocyte depletion to glomerulosclerosis and kidney failure in all progressive glomerular diseases, as well as a study showing that podocyte number per glomerulus decreases with age.^28^ Further, the reduction in podocyte density associated with increased glomerular volume in the retained kidney after a nephrectomy is not associated with an increase in podocyte number per glomerulus as would have been expected if podocytes could be replenished.^42^

We acknowledge that there are potential limitations of our study. The stereological calculations were based on modeling glomerular tufts and capsules as spheres. The PEC densities were underestimated because the vascular and tubular poles of the glomerulus lack PECs and this was not accounted for in the calculations. However, these sources of bias likely affected all study participants to a similar extent and thus would be unlikely to cause a differential bias in the associations detected. The findings of this study are most applicable to persons who undergo a radical nephrectomy for tumor, which would result in glomerular hyperfiltration in the remaining kidney. The association of progressive CKD with podometrics may differ in 2 kidney populations where there no added stress of compensatory glomerular hyperfiltration with the loss of a kidney.

In conclusion, a lower podocytes number per glomerulus is an important predictor of progressive CKD in patients who have undergone a radical nephrectomy. Importantly, this association was unrelated to glomerular hypertrophy. These findings highlight the key role of adequate podocyte number rather than just adequate podocyte density in maintaining glomerular health and suggest the need for further research to explore treatments that can prevent podocyte loss, particularly in high-risk patient populations.

## Disclosure

All the authors declared no competing interests.

### Funding

This study was supported with funding from the 10.13039/100000002National Institutes of Health, 10.13039/100000062National Institute of Diabetes and Digestive and Kidney Diseases (R01 DK090358).

## Data Availability Statement

The data used for the present study are from patient records. Clinical data can only be shared with a data use agreement and Mayo Clinic Institutional Review Board approval.

## Author Contributions

Conceptualization was by AD and ADR. Data curation was by AD, AAS, SKF, AF, and MK. Formal analysis was by AD and AFM. Funding acquisition was by ADR. The investigation was by AD, MPA, JBH, AP, SJS, and ADR. The methodology for podometrics and PECometric estimations from histologic sections were by RW. Resources were by ADR. Supervision was by ADR. Visualization was by AD.

## Supplementary Material

Supplementary File (PDF) **Supplementary Methods.** WT1, Glepp1, and Pax8 immunohistochemical staining. **Figure S1.** An example of a WT1-stained wedge section used to select glomerular tufts at each of the cortical depths (superficial, mid, and deep). **Figure S2.** Examples of 2 PEC and podocyte nuclei on toluidine blue–stained sections by electron microscopy. **Table S1.** Risk of progressive CKD with podocyte measures, overall and by cortex depth. **Table S2.** Unadjusted risk of progressive CKD with podocyte measures, overall and by cortex depth among all 35 cases and 35 controls, 26 cases and 33 controls patients with no diabetes mellitus, and 30 cases and 35 controls with no diabetic nephropathy. **Table S3.** Risk of progressive CKD (case) versus control after adjusting for each other podometric measurement. STROBE Checklist.

## Footnotes

Supplementary File (PDF)
**Supplementary Methods.** WT1, Glepp1, and Pax8 immunohistochemical staining. **Figure S1.** An example of a WT1-stained wedge section used to select glomerular tufts at each of the cortical depths (superficial, mid, and deep). **Figure S2.** Examples of 2 PEC and podocyte nuclei on toluidine blue–stained sections by electron microscopy. **Table S1.** Risk of progressive CKD with podocyte measures, overall and by cortex depth. **Table S2.** Unadjusted risk of progressive CKD with podocyte measures, overall and by cortex depth among all 35 cases and 35 controls, 26 cases and 33 controls patients with no diabetes mellitus, and 30 cases and 35 controls with no diabetic nephropathy. **Table S3.** Risk of progressive CKD (case) versus control after adjusting for each other podometric measurement. STROBE Checklist.
