# Corrigendum to “Lower Podocyte Number per Glomerulus Associates With Progressive CKD” [*Kidney International Reports* Volume 10, Issue 9, September 2025, Pages 3213-3224]

**DOI:** 10.1016/j.ekir.2026.107139

**Published:** 2026-10-08

**Authors:** Aleksandar Denic, Afsana A. Shaik, Syed Khooshal Fareeduddin, Aperna Fnu, Mahesh Kumar, Aidan F. Mullan, Vidit Sharma, Mariam P. Alexander, Akhilesh Pandey, Roger C. Wiggins, Jeffrey B. Hodgin, Stuart J. Shankland, Andrew D. Rule

**Affiliations:** 1 Division of Nephrology and Hypertension, Mayo Clinic, Rochester, Minnesota, USA; 2 Division of Biomedical Statistics and Informatics, Mayo Clinic, Rochester, Minnesota, USA; 3 Department of Urology, Mayo Clinic, Rochester, Minnesota, USA; 4 Department of Laboratory Medicine and Pathology, Mayo Clinic, Rochester, Minnesota, USA; 5 Manipal Academy of Higher Education, Manipal, Karnataka, India; 6 Division of Nephrology, University of Michigan, Ann Arbor, Michigan, USA; 7 Department of Pathology and Internal Medicine, Division of Nephrology, University of Michigan, Ann Arbor, Michigan, USA; 8 Division of Nephrology, University of Washington, Seattle, Washington, USA; 9 Division of Epidemiology, Mayo Clinic, Rochester, Minnesota, USA

The authors regret errors in the units and an equation reported in the above article. In the Methods, podocyte density should be expressed per μm³ rather than per mm³. In Table 3, podocyte density should be expressed per 10⁶ μm³ rather than per 10⁶ mm³, parietal epithelial cell density should be expressed per μm² rather than per mm², and podocyte cell volume should be expressed in μm³ rather than mm³. In addition, the equation for podocyte density was incorrect. Equation 3 should read: Podocyte density = True podocyte nuclear number / (tissue section thickness × glomerular tuft area).

DOI of original article: https://doi.org/10.1016/j.ekir.2025.06.004

