# Supplementary material for: Lower Podocyte Number per Glomerulus Associates With Progressive CKD
Source: Kidney Int Rep. 2025 Jun 11;10(9):3213–24. doi: 10.1016/j.ekir.2025.06.004 (PMC12446936; doi:10.1016/j.ekir.2025.06.004)
Supplement: Supplementary File (PDF) — Supplementary Methods. WT1, Glepp1, and Pax8 immunohistochemical staining. Figure S1. An example of a WT1-stained wedge section used to select glomerular tufts at each of the cortical depths (superficial, mid, and deep). Figure S2. Examples of 2 PEC and podocyte nuclei on toluidine blue–stained sections by electron microscopy. Table S1. Risk of progressive CKD with podocyte measures, overall and by cortex depth. Table S2. Unadjusted risk of progressive CKD with podocyte measures, overall and by cortex depth among all 35 cases and 35 controls, 26 cases and 33 controls patients with no diabetes mellitus, and 30 cases and 35 controls with no diabetic nephropathy. Table S3. Risk of progressive CKD (case) versus control after adjusting for each other podometric measurement. STROBE Checklist. [file mmc1.pdf]

Supplemental Material

1. Supplemental Methods

WT1, Glepp1 and Pax8 Immunohistochemical Staining

Immunohistochemical staining was performed at the Pathology Research Core (Mayo Clinic, Rochester, MN) using the Leica Bond RX stainer (Leica). Wilm's Tumor (WT1) stain was used to detect podocyte nuclei,[20] Glomerular epithelial protein 1 (Glepp1) was used to detect the podocyte cytoplasm,[22] and paired box gene 8 (Pax8) was used to detect PEC nuclei.[23] Two adjacent tissue sections were cut at 5 microns, mounted on charged slides, and dried overnight. For staining, slides stained for Wilm's Tumor (WT1) were retrieved for 20 minutes using Epitope Retrieval 2 (EDTA; Leica) and incubated in Protein Block (Dako) for 5 minutes. All primary antibodies were diluted in Background Reducing Diluent (Dako). The WT1 primary antibody (clone: WT49; Leica) was diluted to 1:100 and incubated for 45 minutes. The Pax8 primary antibody (clone: SP348, Abcam) was diluted to 1:300 and incubated for 45 minutes. The detection system used was Polymer Refine Detection System (Leica). This system includes hydrogen peroxidase block, post primary and polymer reagent, and DAB. Immunostaining visualization was achieved by incubating slides for WT1 and Pax8 for 20 minutes. Slides were counterstained for 5 minutes using a 1:1 mixture of Schmidt hematoxylin (Mayo DLMP Preparation and Processing Laboratory) and molecular biology grade water. Once the immunochemistry process was completed, slides were removed from the stainer and rinsed in tap water for 3 minutes. Slides were dehydrated in increasing concentrations of ethyl alcohol and cleared in 3 changes of xylene prior to permanent cover slipping in xylene-based medium. Slides stained with WT1, and Pax8 were imaged at a 40x resolution using the GT450, brightfield whole slide scanner from Leica. Once slides were scanned, they were soaked in Xylene for coverslip removal. Once the coverslips came off the slides, they were hydrated in decreasing concentrations of ethyl alcohol and rinse in diH2O for 5 minutes. Slides were loaded wet, using PBS, onto the Leica BOND RX for Glepp1 (PTPRO) staining. Slides were retrieved for 10 minutes using Epitope Retrieval 2 and incubated for five minutes in Protein Block. The Glepp1 primary antibody (clone: 2F2B4, Protein Tech) was diluted to 1:5000 and incubated for 15 minutes. The detection system, as well as the final steps, were the same as described above. Immunostaining visualization was achieved by incubating the slides for 15 minutes in DAB and DAB buffer. Slides double stained with WT/Glepp1 were imaged again.

2. Supplemental Tables

Supplemental Table 1. Risk of progressive CKD with podocyte measures, overall and by cortex depth.

**Podometrics (all per SD)**	**Unadjusted**	**Adjusted for glomerular tuft volume and nephrosclerosis measures***	**Adjusted for clinical characteristics****
	**OR (95% CI)**	**OR (95% CI)**	**OR (95% CI)**
**Overall cortex**			
Podocyte number per glomerulus	**0.39 (0.19, 0.79)**	**0.30 (0.10, 0.91)**	**0.30 (0.12, 0.78)**
Podocyte density	**0.54 (0.30, 0.96)**	**0.47 (0.22, 1.00)**	0.51 (0.23, 1.11)
Podocyte cell volume	1.48 (0.87, 2.52)	1.54 (0.72, 3.27)	1.47 (0.67, 3.22)
PEC number per glomerulus	0.87 (0.54, 1.40)	0.78 (0.37, 1.65)	0.70 (0.36, 1.35)
PEC density	0.69 (0.42, 1.15)	0.54 (0.25, 1.15)	0.69 (0.36, 1.33)
**Superficial cortex**			
Podocyte number per glomerulus	**0.54 (0.29, 0.98)**	0.51 (0.22, 1.16)	0.51 (0.22, 1.20)
Podocyte density	**0.55 (0.31, 0.98)**	0.57 (0.30, 1.11)	0.37 (0.14, 1.01)
Podocyte cell volume	1.46 (0.86, 2.45)	1.38 (0.66, 2.89)	1.57 (0.69, 3.58)
PEC number per glomerulus	1.13 (0.71, 1.81)	1.30 (0.57, 2.96)	1.24 (0.67, 2.31)
PEC density	0.82 (0.49, 1.35)	0.79 (0.38, 1.64)	0.89 (0.45, 1.76)
**Middle cortex**			
Podocyte number per glomerulus	**0.43 (0.21, 0.89)**	**0.28 (0.08, 0.91)**	**0.31 (0.13, 0.78)**
Podocyte density	**0.51 (0.28, 0.96)**	**0.38 (0.15, 0.95)**	0.53 (0.24, 1.15)
Podocyte cell volume	1.44 (0.82, 2.55)	1.67 (0.70, 4.02)	1.36 (0.64, 2.91)
PEC number per glomerulus	0.79 (0.49, 1.26)	0.63 (0.32, 1.27)	0.58 (0.29, 1.17)
PEC density	0.68 (0.42, 1.11)	**0.47 (0.22, 0.99)**	0.69 (0.37, 1.28)
**Deep cortex**			
Podocyte number per glomerulus	**0.34 (0.16, 0.72)**	**0.32 (0.11, 0.94)**	**0.30 (0.12, 0.75)**
Podocyte density	**0.56 (0.32, 0.98)**	**0.47 (0.23, 0.95)**	0.64 (0.32, 1.25)
Podocyte cell volume	1.65 (0.93, 2.94)	1.81 (0.77, 4.25)	1.59 (0.69, 3.64)
PEC number per glomerulus	0.81 (0.47, 1.40)	0.76 (0.33, 1.75)	0.54 (0.25, 1.15)
PEC density	0.66 (0.38, 1.12)	0.54 (0.26, 1.13)	0.61 (0.31, 1.20)

*Nephrosclerosis measures: %GSG, %IFTA, IFTA foci density, and %artery luminal stenosis.
**Diabetes mellitus, BMI, hypertension, baseline eGFR, proteinuria.

Supplemental Table 2. Unadjusted Risk of progressive CKD with podocyte measures, overall and by cortex depth among all 35 cases and 35 controls, 26 cases and 33 controls patients with no diabetes mellitus, and 30 cases and 35 controls with no diabetic nephropathy.

Podometrics (all per SD)	No exclusions	Excluding diabetes mellitus	Excluding diabetic nephropathy
	OR (95% CI)	OR (95% CI)	OR (95% CI)
**Overall cortex**			
Podocyte number per glomerulus	**0.39 (0.19, 0.79)**	**0.57 (0.31-0.99)**	**0.49 (0.25-0.86)**
Podocyte density	**0.54 (0.30, 0.96)**	0.65 (0.36-1.11)	0.60 (0.33-1.02)
Podocyte cell volume	1.48 (0.87, 2.52)	1.22 (0.72-2.12)	1.28 (0.74-2.32)
PEC number per glomerulus	0.87 (0.54, 1.40)	0.87 (0.50-1.46)	0.84 (0.50-1.37)
PEC density	0.69 (0.42, 1.15)	0.72 (0.41-1.23)	0.71 (0.41-1.17)
**Superficial cortex**			
Podocyte number per glomerulus	**0.54 (0.29, 0.98)**	0.62 (0.34-1.05)	**0.58 (0.33-0.98)**
Podocyte density	**0.55 (0.31, 0.98)**	0.71 (0.40-1.21)	0.65 (0.37-1.09)
Podocyte cell volume	1.46 (0.86, 2.45)	1.14 (0.67-1.97)	1.26 (0.75-2.20)
PEC number per glomerulus	1.13 (0.71, 1.81)	1.12 (0.66-1.91)	1.13 (0.69-1.88)
PEC density	0.82 (0.49, 1.35)	0.91 (0.53-1.53)	0.87 (0.52-1.43)
**Middle cortex**			
Podocyte number per glomerulus	**0.43 (0.21, 0.89)**	0.65 (0.36-1.12)	**0.54 (0.28-0.93)**
Podocyte density	**0.51 (0.28, 0.96)**	0.60 (0.32-1.03)	**0.57 (0.31-0.98)**
Podocyte cell volume	1.44 (0.82, 2.55)	1.28 (0.76-2.32)	1.27 (0.75-2.33)
PEC number per glomerulus	0.79 (0.49, 1.26)	0.79 (0.46-1.33)	0.72 (0.42-1.19)
PEC density	0.68 (0.42, 1.11)	0.66 (0.37-1.12)	0.63 (0.36-1.06)
**Deep cortex**			
Podocyte number per glomerulus	**0.34 (0.16, 0.72)**	**0.51 (0.26-0.90)**	**0.46 (0.24-0.81)**
Podocyte density	**0.56 (0.32, 0.98)**	0.65 (0.35-1.13)	0.61 (0.33-1.04)
Podocyte cell volume	1.65 (0.93, 2.94)	1.31 (0.78-2.31)	1.43 (0.76-2.85)
PEC number per glomerulus	0.81 (0.47, 1.40)	0.83 (0.48-1.39)	0.83 (0.50-1.36)
PEC density	0.66 (0.38, 1.12)	0.72 (0.41-1.21)	0.72 (0.43-1.19)

Supplemental Table 3. Risk of progressive CKD (case) versus control after adjusting for each other podometric measurement.

Podometrics (all per SD)	Adjusted for each other podometric measure OR (95% CI)
**Model 1**	
Podocyte number per glomerulus	**0.34 (0.13, 0.89)**
Podocyte cell volume	0.95 (0.45, 2.01)
PEC density	1.12 (0.56, 2.26)
**Model 2**	
Podocyte density	0.42 (0.15, 1.20)
Podocyte cell volume	0.75 (0.31, 1.82)
PEC density	1.01 (0.53, 1.93)

Supplemental Figures

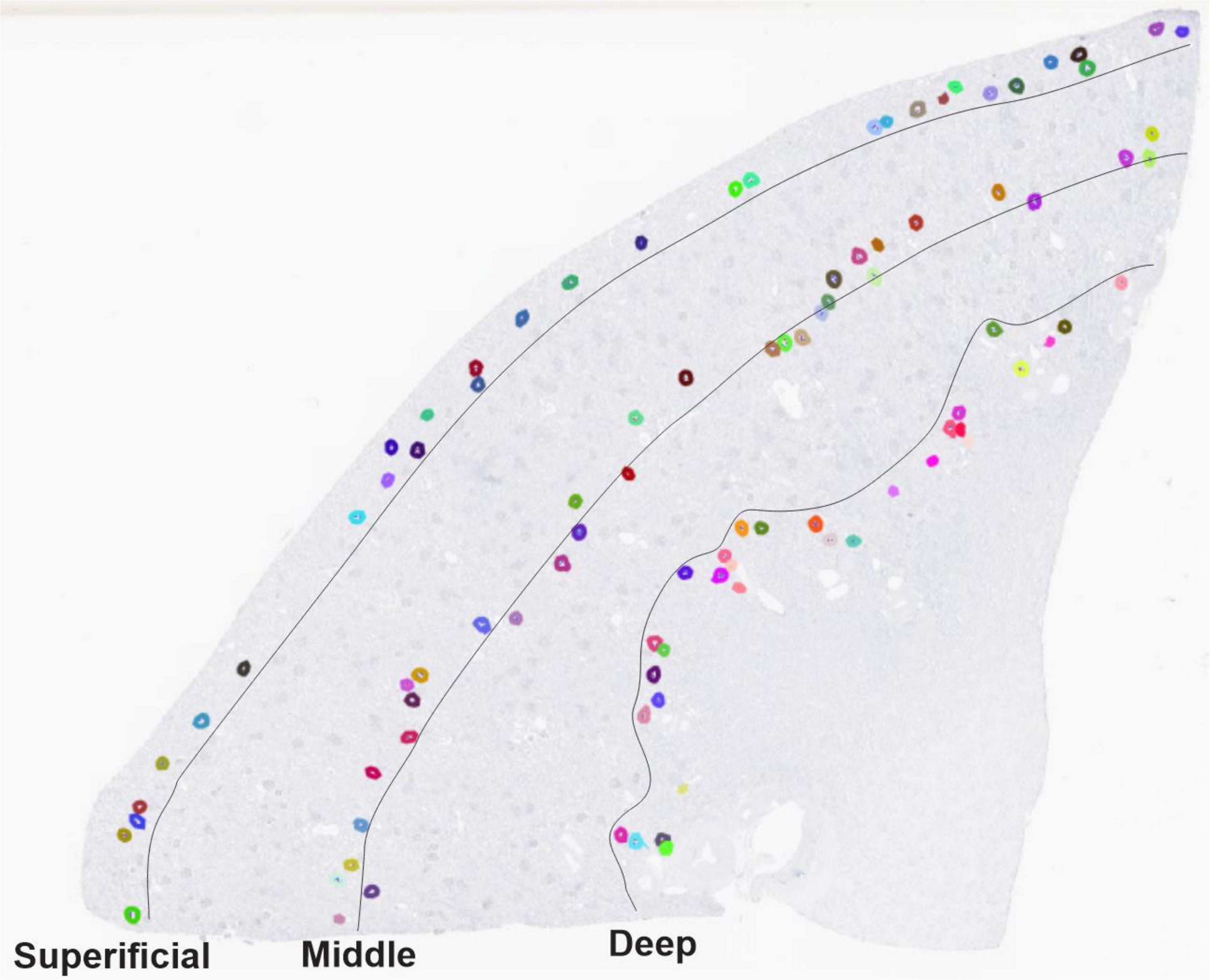

Supplemental Figure 1. An example of a WT1-stained wedge section used to select glomerular tufts at each of the cortical depths (superficial, meed and deep). Then, WT1-staned podocytes were quantified in QuPath.

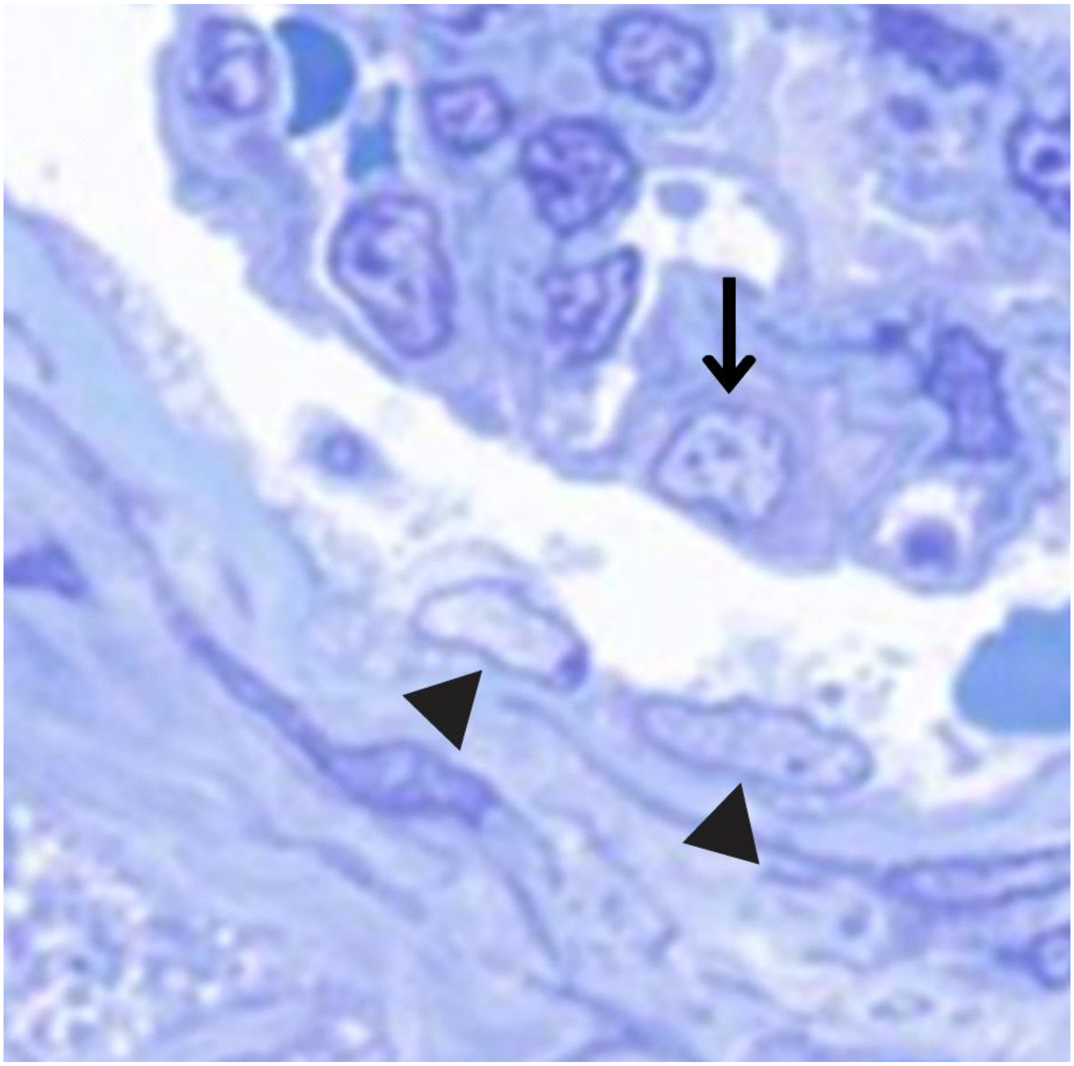

Supplemental Figure 2. Examples of two PEC and podocyte nuclei on toluidine blue stained sections by electron microscopy. Black arrowheads point to the more flattened PEC nuclei compared to the rounder podocyte nuclei (black arrow).

STROBE Statement—checklist of items that should be included in reports of observational studies

	Item No.	Recommendation	Page No.	Relevant text from manuscript
**Title and abstract**	1	(*a*) Indicate the study's design with a commonly used term in the title or the abstract	1	
		(*b*) Provide in the abstract an informative and balanced summary of what was done and what was found	2	
**Introduction**				
Background/rationale	2	Explain the scientific background and rationale for the investigation being reported	3	
Objectives	3	State specific objectives, including any prespecified hypotheses	3	The goal of this study was to determine if podometric measures associated with the subsequent development of progressive CKD
**Methods**				
Study design	4	Present key elements of study design early in the paper	4	cases with progressive CKD were identified… matched controls without progressive CKD were identified
Setting	5	Describe the setting, locations, and relevant dates, including periods of recruitment, exposure, follow-up, and data collection	4	We studied patients in the Aging Kidney Anatomy study[1]
Participants	6	(*a*) *Cohort study*—Give the eligibility criteria, and the sources and methods of selection of participants. Describe methods of follow-up<br><br>*Case-control study*—Give the eligibility criteria, and the sources and methods of case ascertainment and control selection. Give the rationale for the choice of cases and controls<br><br>*Cross-sectional study*—Give the eligibility criteria, and the sources and methods of selection of participants	4	Figure 2

		(*b*) *Cohort study*—For matched studies, give matching criteria and number of exposed and unexposed<br><br>*Case-control study*—For matched studies, give matching criteria and the number of controls per case		
Variables	7	Clearly define all outcomes, exposures, predictors, potential confounders, and effect modifiers. Give diagnostic criteria, if applicable	4-6	
Data sources/ measurement	8*	For each variable of interest, give sources of data and details of methods of assessment (measurement). Describe comparability of assessment methods if there is more than one group	4-6 Supplemental methods	PEC nuclear shape coefficient, Podometrics, Converting density to number, morphometrics
Bias	9	Describe any efforts to address potential sources of bias	4-5	Measurement bias addressed by calculating PEC nuclear shape coefficient and using oil immersion microscopy to measure section thickness.
Study size	10	Explain how the study size was arrived at	4	Figure 2

Continued on next page

Quantitative variables	11	Explain how quantitative variables were handled in the analyses. If applicable, describe which groupings were chosen and why	4-6	Clinical, podometrics, and histologic morphology grouping described
Statistical methods	12	(*a*) Describe all statistical methods, including those used to control for confounding	6	Logistic regression with adjusted analyses
		(*b*) Describe any methods used to examine subgroups and interactions	6	Analyses overall and by each depth
		(*c*) Explain how missing data were addressed	N/A	Nephron number missing in some but this covariate was not used in the adjusted analyses.
		(*d*) *Cohort study*—If applicable, explain how loss to follow-up was addressed<br>*Case-control study*—If applicable, explain how matching of cases and controls was addressed<br>*Cross-sectional study*—If applicable, describe analytical methods taking account of sampling strategy	4	Age, sex, and follow-up time matched
		(*e*) Describe any sensitivity analyses	none	
**Results**				
Participants	13*	(a) Report numbers of individuals at each stage of study—eg numbers potentially eligible, examined for eligibility, confirmed eligible, included in the study, completing follow-up, and analysed	Figure 2	35 cases and 35 controls
		(b) Give reasons for non-participation at each stage	Figure 2	
		(c) Consider use of a flow diagram	Figure 2	
Descriptive data	14*	(a) Give characteristics of study participants (eg demographic, clinical, social) and information on exposures and potential confounders	Table 1	
		(b) Indicate number of participants with missing data for each variable of interest	Table 1 footnote	Nephron number missing in some patients.
		(c) *Cohort study*—Summarise follow-up time (eg, average and total amount)	Table 1	
Outcome data	15*	*Cohort study*—Report numbers of outcome events or summary measures over time		
		Case-control study—Report numbers in each exposure category, or summary measures of exposure	Table 1	

		Cross-sectional study—Report numbers of outcome events or summary measures	
Main results	16	(*a*) Give unadjusted estimates and, if applicable, confounder-adjusted estimates and their precision (eg, 95% confidence interval). Make clear which confounders were adjusted for and why they were included	Tables 3&4
		(*b*) Report category boundaries when continuous variables were categorized	n/a
		(*c*) If relevant, consider translating estimates of relative risk into absolute risk for a meaningful time period	n/a

Continued on next page

Other analyses	17	Report other analyses done—eg analyses of subgroups and interactions, and sensitivity analyses	Tables 2 and 5	Cross-sectional correlations
**Discussion**				
Key results	18	Summarise key results with reference to study objectives	7	1st paragraph
Limitations	19	Discuss limitations of the study, taking into account sources of potential bias or imprecision. Discuss both direction and magnitude of any potential bias	9	
Interpretation	20	Give a cautious overall interpretation of results considering objectives, limitations, multiplicity of analyses, results from similar studies, and other relevant evidence	7-9	
Generalisability	21	Discuss the generalisability (external validity) of the study results	9	Association… may differ in 2 kidney populations
**Other information**				
Funding	22	Give the source of funding and the role of the funders for the present study and, if applicable, for the original study on which the present article is based	9	NIH/NIDDK

*Give information separately for cases and controls in case-control studies and, if applicable, for exposed and unexposed groups in cohort and cross-sectional studies.

Note: An Explanation and Elaboration article discusses each checklist item and gives methodological background and published examples of transparent reporting. The STROBE checklist is best used in conjunction with this article (freely available on the Web sites of PLoS Medicine at http://www.plosmedicine.org/, Annals of Internal Medicine at http://www.annals.org/, and Epidemiology at http://www.epidem.com/). Information on the STROBE Initiative is available at www.strobe-statement.org.
